# Loneliness, Malnutrition and Change in Subjective Age among Older Adults during COVID-19 Pandemic

**DOI:** 10.3390/ijerph18010106

**Published:** 2020-12-26

**Authors:** Adi Vitman Schorr, Itamar Yehuda, Snait Tamir

**Affiliations:** 1 Shamir Research Institute, Haifa University, Katsrin 1290000, Israel; itamary@telhai.ac.il; 2 Faculty of Science and Technology, Tel-Hai College, Upper Galilee 1220800, Israel; snait@telhai.ac.il; 3 Laboratory of Human Health and Nutrition Sciences, MIGAL-Galilee Research Institute, Kiryat-Shmona 11016, Israel

**Keywords:** feelings of loneliness, subjective age, malnutrition, COVID-19, older adults

## Abstract

*Objectives:* We examined the effect of loneliness and the role of two mediating factors, depressive symptoms and malnutrition on subjective age among older adults during the 2020 COVID-19 pandemic, and explored how the pandemic is affecting subjective age. *Design:* A convenience sample of 201 older adults aged 65 and over was interviewed. Using bootstrapping, we tested the strength and significance of the indirect effect of depressive symptoms and malnutrition (mediators) on the relationship between feelings of loneliness and subjective age. *Results:* The relationship between feelings of loneliness and subjective age during the COVID-19 pandemic was mediated by malnutrition, but not by depressive symptoms. In addition, the participants felt older during the COVID-19 pandemic compared with the preceding period. *Conclusions*: An association was found among feelings of loneliness, malnutrition, and subjective age. To overcome these feelings in times of crisis like the pandemic, it is essential to develop new communication methods (technologies for managing and addressing the needs of the older population; technologies to encourage social engagement, and technologies for managing and providing remote medical services) for and with older adults that are effective in reducing loneliness, and to promote good nutrition. Possible practical solutions include new social network technologies for reducing loneliness combined with continued reliance on phone communication as an intervention of psychological support to promote a healthy lifestyle and prevent malnutrition.

## 1. Introduction

### 1.1. Subjective Age of Older Adults

Subjective age refers to the degree to which people feel younger or older than their chronological age [1]. Like chronological age, subjective age contributes to a variety of developmental outcomes [2]. People who feel younger than their chronological age are usually better off than those who feel their actual age or older [3]. For instance, the outcomes of one of the only meta-analyses that has investigated the longitudinal effect of subjective age on future health and longevity among adults (average age 57–85 years) revealed that feeling younger is connected with improved physical health and longevity [4]. Furthermore, a correlation has been found between younger subjective age and important developmental processes, such as enhanced subjective wellbeing [5], better cognition test performance [6], and having fewer depressive symptoms [7]. Research has found that feeling younger than one’s chronological age is associated with higher levels of subjective wellbeing [5], greater life satisfaction [8,9], and more positive affect [5,10]. Younger subjective age is also associated with having a sense of meaning in life, higher levels of optimism, and more successful aging [8]. Similarly, feeling younger correlates with a decreased likelihood of experiencing a major episode of depression [11] and reduced symptoms of depression [12].

In another meta-analysis, women reported a younger subjective age compared to that reported by men [13]. Later studies have provided more evidence that being a woman is correlated with younger subjective age [5,14,15]; however, others have not found this correlation [6,16,17,18]. The impact of loneliness on subjective age is not clear; one study found that a decrease in loneliness resulted in a decrease in subjective age, but, changes in objective social indicators did not predict changes in subjective age [19].

### 1.2. Loneliness in Older Adults

The definition of loneliness is the gap between real and wished for social relationships [20,21]. Similar to subjective age, loneliness is a subjective concept and not an objective social manifestation [20]. Although loneliness can be connected with objective aspects of the social network including the number and frequency of actual social contacts, it is not synonymous with these aspects and it still represents the qualitative elements of relationships [22].

With a few exceptions, the majority of past research has emphasized the role of loneliness as a strong predictor of morbidity and mortality [23,24]. A considerable body of research has pointed out that a high level of loneliness is a major risk factor for cardiovascular diseases [25], disability [26], poor sleep hygiene [27], impaired cognition [28], and impaired physical functioning [22]. These negative effects of loneliness might explain the possible association between feelings of loneliness and subjective age, and in contrast, the negative correlation between size of friendship network and self-perception of age (for example, the finding that women who felt themselves younger than their actual age had larger friendship networks [29]. Moreover, a recent study suggested a plausible causal model of loneliness leading to morbidity and mortality, and found evidence of mediation by subjective health, depressive symptoms, and functional limitations [30].

Although objective indicators of social relationships also predict health and well-being [31,32], these are generally thought to exert a somewhat smaller effect in comparison to loneliness. However, there is general consent that loneliness increases with age among older adults [33]. This is not surprising given the many objective losses that take place in advanced age [33]. Objective losses include, for example, retirement because it is often associated with the narrowing of one’s social network or the death of a spouse, siblings, and close friends, which also result in reduced social contact [34,35].

### 1.3. Mediating Factors

In addition to the possible direct correlation between loneliness and subjective age, during a crisis like the Coronavirus disease (COVID-19) pandemic, feelings of loneliness can have far-reaching implications for the life of older adults, which could shed light on some of the effects of feeling lonely on subjective age during a crisis. Two specific mediators are likely to be at play—depressive symptoms and malnutrition.

#### 1.3.1. Depressive Symptoms

According to the 2001 World Health Organization (WHO) Global Burden of Disease Study, depression is a serious public health problem among individuals, families, and societies throughout the world. The WHO estimated that depression was the fourth leading contributor to the global burden of disease in 2000, as measured by disability-adjusted life years [36]. Although depression rates are generally lower among older adults (5.4%) compared to middle-aged (9.8%) and younger (7.4%) adults, the rate among seniors in the United States has continued to rise in recent decades [37,38,39]. In addition, the reported rates of depression in the older population of the US may be underestimated. This is because depressive symptoms can be masked as physical complaints or initially appear to be cognitive impairments in this age group, moreover, the stigma of mental illness may inhibit depressed older adults from seeking treatment.

Depression is one of the negative health outcomes linked to loneliness as well as disability, and cognitive decline [40,41,42,43]. For example, a study in Ohio retirement communities found that older individuals who reported feeling lonely had significantly higher depression scores [44]. However, the experiences and consequences of loneliness may vary greatly. Moreover, because subjective age reflects self-perception, it is also related to many psychological factors among older adults. Research has shown that people who feel younger than their chronological age tend to be mentally healthy and have fewer psychological problems [11,12]. In addition, younger subjective age has been associated with less stress [1], fewer depressive symptoms [45,46], and strong mastery beliefs [45,47].

#### 1.3.2. Malnutrition

Malnutrition is defined as a state in which a deficiency, excess, or imbalance of energy, protein, and other nutrients causes measurable adverse effects on tissue and body form (body shape, size, and composition), function, and clinical outcomes [48]. It is more prevalent as age increases [49,50,51]. The etiology of malnutrition is multifactorial; adverse physiological, psychological, and social causes of malnutrition in older adults are consistently reported in the literature [52]. Aging is accompanied by physiological changes that can negatively impact nutritional status, for example, sensory impairment may result in reduced appetite and poor oral health, and dental problems can lead to difficulty chewing, inflammation, and a monotonous, poor-quality diet. Progressive loss of vision and hearing may also limit mobility and affect the ability to shop for food and prepare meals [53,54].

In addition to loneliness and depression, other psychosocial and social changes characteristic of older adults, such as cognitive impairment, heavy use of medication, periods of lengthy hospitalization, retirement from paid work, bereavement, and increasing frailty can also contribute to poor nutritional status [54,55]. These factors affect the ability of older adults to meet dietary needs or to digest, absorb, utilize, or excrete nutrients that are ingested, leading to reduced energy intake and lean body mass. This, in turn, may result in a reduced metabolic rate, a corresponding decline in total energy expenditure, and potentially to malnutrition [56,57,58]. Thus, malnutrition, like other unhealthy outcomes of old age, may also be associated with subjective age [45].

## 2. The Current Study

In response to the COVID-19 pandemic that began in 2019, a policy of social distancing was initiated worldwide. Although circumstances necessitate such extreme measures, social isolation presents a risk for adverse health effects [59]. Older adults who are at greater risk for COVID-19 health complications are likely to remain in strict self-isolation longer than other age groups; therefore, the effects of isolation and ensuing loneliness may be especially severe for them [60]. Loneliness reflects subjective distress resulting from a discrepancy between desired and perceived social relationships. Unfortunately, it causes a host of poor outcomes, such as depression, anxiety, physical morbidity, and mortality, and might also correlate with older subjective age, which is associated with further health risks [4].

The current study assessed the status of feelings of loneliness and subjective age among older adults during the COVID-19 pandemic. We identified the factors underlying the association between the two and explored the role of two potential mediating factors—depressive symptoms and malnutrition. The study also explored how subjective age is changing during the COVID-19 pandemic.

In light of the literature reviewed, we posited three hypotheses:

1. In comparison with the period before the pandemic, older adults feel older in age during the pandemic.
2. Feelings of loneliness are associated with subjective age during times of crisis.
3. Feelings of loneliness are indirectly associated with subjective age during crises through depressive symptoms and malnutrition; lonely older adults feel older during crises, and this is associated with higher levels of depressive symptoms and malnutrition.

## 3. Method

### 3.1. Study Design and Participants

This was a cross-sectional study of a convenience sample of 201 older adults aged 65 and over, who represent the two main ethnic groups living in Israel, Jews and Arabs. Inclusion criteria were age 65 and over and the ability to speak and understand (but not necessarily read) Hebrew or Arabic. The refusal rate was 21 percent.

### 3.2. Procedures

The Research Ethics Committee of the college at which the research took place approved the study. Those who agreed to participate received an explanation about the study and their right to withdraw at any time without penalty. Strict confidentiality was maintained. Professional interviewers collected the data in telephone interviews (in compliance with pandemic-related restrictions), using appropriate translated, validated, and structured questionnaires. The participants were recruited randomly. The final sample comprised 201 participants: 100 Jews and 101 Arabs. The data were collected in April and May 2020.

### 3.3. Measures

#### 3.3.1. Independent Variable: Loneliness

Loneliness was measured by a single direct question: “Do you sometimes feel lonely?” with four options: never, seldom, sometimes, often.

#### 3.3.2. Dependent Variable: Subjective Age during a Crisis (the COVID-19 Pandemic).

Subjective age was measured by two direct questions: “On a scale from 0 to 5, how old do you feel?” The six options were on a scale between 0 = younger than my actual age, and 5 = older than my actual age. The participants were asked to refer to the time before the COVID-19 pandemic and the time during the pandemic.

### 3.4. Mediators

#### 3.4.1. Depressive Symptoms

Depressive symptoms were measured using the GDS (Geriatrics Depression Scale; [61]). This questionnaire was chosen because it is a simple and reliable tool that allows the examination of depressive symptoms among older adults without requiring a professional interviewer. The instrument is composed of 15 items in a yes (1)/no (2) response format. The internal reliability (Cronbach’s alpha) was 0.80.

#### 3.4.2. Malnutrition

Malnutrition was measured using the NSI (Determine Nutrition Screening Initiative) instrument, developed by the American Diabetes Association, the American Family Doctors Association, and the National Council of Old Age to detect older adults at risk for malnutrition. The questionnaire is composed of 10 items, in a yes (with changing scores)/no (0) response format.

### 3.5. Covariates

We controlled for the socioeconomic variables of gender (dichotomous; 0 = male, 1 = female), age, years of education (both continuous), and marital status (with partner = 1, or without a partner = 0).

All the instruments were translated into Hebrew and Arabic by bilingual translators. The complete questionnaire underwent a pilot test. The questionnaire took approximately 15 min to complete, the verbal instructions were comprehensible, and there was no need for further changes prior to administering it.

### 3.6. Data Analyses

The data analyses included four stages. In the first stage, descriptive statistics were employed to calculate the means and standard deviations of the continuous variables and the percentage and frequency of the categorical variables. In the second stage, in order to test the first hypothesis regarding the difference in subjective age before and during the COVID-19 pandemic, a paired *t*-test was used. In the third stage, bivariate analyses were performed to examine the association between subjective age and the independent variable, mediator variables, and socioeconomic variables using an independent *t*-test, one-way ANOVA, and Pearson or Spearman correlation tests.

In the fourth stage, mediation analyses were then computed, and the selected mediators (depressive symptoms and malnutrition) were entered to test the components of the mediation model (Model 4). The bootstrapping method was used to assess the indirect effects of the mediation model [62,63]. Thus, the mediation model was examined by directly testing the significance of the indirect effect of the independent variable (IV; feelings of loneliness) on the dependent variable (DV; subjective age) through the mediators (MeV; depressive symptoms and malnutrition), while controlling for background variables that had been identified in the bivariate analyses as significant.

This method is based on regression analysis and calculates the direct effect (weight *C’*, with a mediator), total effect (*C*, without mediator) and indirect effects (*a × b* weights) of an independent variable on a dependent variable. The total and specific indirect effects were calculated through bootstrapping, set at 5000 samples. Confidence intervals were calculated using this method by sorting the lowest to highest of these samples, yielding a 95-percentile confidence interval (if the number 0 falls within the confidence intervals, the tested effect is nonsignificant).

All analyses were run using SPSS 25.0 with the PROCESS statistical program [62]. All estimated effects reported by PROCESS are unstandardized regression coefficients.

## 4. Results

Table 1 presents the background variables, the descriptive statistics for the mediators and the independent and dependent variables of the sample. The majority of the participants were women, aged between 65 and 95 (mean = 74.3, *SD =* 6.3) with moderate health status (Mean = 3.61, *SD =* 1.15). Their education ranged from 6 to 21 years (Mean = 10.0, *SD =* 4.0) and most had a partner (71.1%). About 50% were Arabs and the others, Jews. No differences were found between the two ethnic groups (data not shown).

The feelings of loneliness of the participants were medium (Mean = 2.2, *SD* = 1.1); depressive symptoms were fairly low (Mean = 4.8, *SD* = 3.2); malnutrition was low-to-medium (Mean = 6.8, *SD* = 4.3) and subjective age during the COVID-19 pandemic was medium (Mean = 2.94, *SD* = 1.73).

The first hypothesis is supported by the results of the paired *t*-test. A difference was indicated between the two times, and the participants felt older during the pandemic (see Table 2).

The results of the bivariate tests of the association between the research variables and subjective age during the COVID-19 pandemic reveal that the only demographic variable that correlated significantly with subjective age during the crisis was marital status (having a partner correlated positively with lower subjective age, see Table 3).

The results reveal that all the independent and mediation variables were significantly related to the dependent variable. High levels of loneliness, depressive symptoms, and malnutrition correlated positively with subjective age during the pandemic.

### The Mediation Analyses

**Figure 1:**
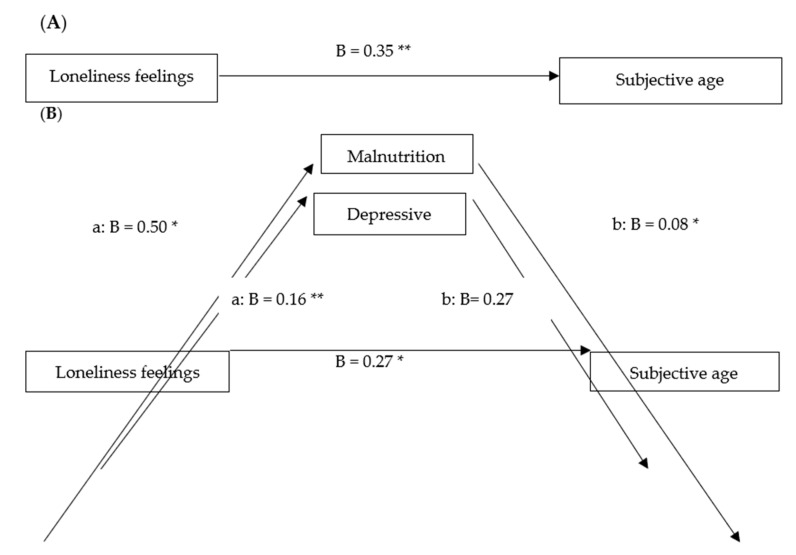
Mediator model depicting direct and indirect effects of feelings loneliness on subjective age, controlling for background variables. Graphic (**A**) depicts the total effect of loneliness on subjective age. Graphic (**B**) depicts the direct effect of loneliness on subjective age, after including mediators and controlling for all background variables. Values represent unstandardized regression coefficients.

Using the PROCESS model 4 [62,63], we tested the second and third hypotheses that during the crisis, feelings of loneliness would be associated directly with subjective age, and indirectly associated with subjective age through depressive symptoms and malnutrition, controlling for covariates (see Table 4 and Figure 1). The results indicate a significant total direct effect (*path c*; without mediators) of loneliness on subjective age, and a significant indirect effect through malnutrition as a mediator. No indirect connection was found between the second mediator (depressive symptoms) and subjective age. No significant associations were found between the background variables and subjective age.

## 5. Discussion

The present research was conducted during the COVID-19 pandemic. During this crisis, older adults are being considered a population at risk; therefore, people of this age are expected to maintain physical distance and reduce the frequency of leaving their home as much as possible [64]. In light of the loneliness potentially caused by social and physical distancing, the purpose of this study was to examine the possible connection between loneliness and subjective age during the COVID-19 pandemic, and to investigate whether loneliness was indirectly associated with subjective age through depressive symptoms and malnutrition. Finally, an attempt was made to determine whether older adults, at a time when their age puts them at risk in terms of physical and mental health, felt older than their chronological age [1].

The research findings confirm the first hypothesis: the older adults’ subjective age was higher at this time of crisis compared with a less stressful time. This result is consistent with those of other studies [1,65] that showed that older adults had a higher subjective age in times of crisis, distress, and stress. There are several possible explanations of this finding. First, the influence of stress and age are interactive. Psychological stress can both imitate and worsen the effects of aging, with older adults usually showing greater immunological impairment and stress than younger adults [65]. Previous studies have shown that older subjective age is related to higher levels of stress [66,67]. This could be particularly true for daily stressors, which, if they accumulate, create persistent irritation and overload that may result in more serious stress reactions [68,69] such as less energy. The resulting feeling (or actual state) of exhaustion is consistent with feeling older.

With regard to the second and third hypotheses, the results indicate a direct and positive connection between feelings of loneliness and subjective age; the lonelier the participants, the higher was their subjective age. This finding is also consistent with the literature [19,29]. The size of one’s social network, the satisfaction related to it, and feeling part of the social network have been found to be associated with subjective age [29,70]. Researchers have also found that the connection between loneliness and subjective age is bidirectional. Having a large social network is associated with younger subjective age; older adults who feel younger tend to be friendlier than their cohorts who feel older, and conversely, feeling older than one’s chronological age may limit the desire and capacity for maintaining social relationships, which may reflect compromised physical and emotional well-being [29]. The examination of two variables that might mediate the direct connection between loneliness and subjective age confirmed the effect of malnutrition, but not depressive symptoms.

Previous research has addressed the connection between loneliness and malnutrition, and has found that loneliness may affect appetite and nutrient intake through a decline in mood, physical functioning, or cognition [71]. These, in turn, combined with the difficulty of eating alone and changes in social status, particularly due to the loss of a spouse or friends of the same age group, can further inhibit appetite [72]. In comparison, eating with others can help prevent malnutrition. It increases caloric intake, is related to healthier food habits [73,74], and maintains the motivation of older adults to eat and cook, providing them with opportunities for social interaction and connectedness [75]. Another possible explanation is the association between deconditioning and loneliness, deconditioning contributes to frailty and fear of falling. The fear of falling could also contribute to avoiding grocery shopping and a diminished desire to stand while preparing meals. To the best of our knowledge, the finding of a correlation between malnutrition and subjective age is new. It can be explained by the findings of previous research that in older adults, a young subjective age is associated with good health, functional health, and wellbeing [4,45]. Moreover, a comparison between unobserved (blood pressure and telomere length) and observed (grip strength, expiratory flow, and waist circumference) health measures found that the observed health measures were connected to younger subjective age [2]. Against this background, it can be assumed that inadequate nutrition that leads to malnutrition could become an observed health problem that influences functional health. These findings help explain the connection between malnutrition and subjective age.

## 6. Conclusions and Implications

The results of the present study indicate that there is an association between feelings of loneliness and malnutrition and subjective age. The primary conclusion is that loneliness affects both malnutrition and subjective age (which, as noted, is associated with numerous negative psychological and physical outcomes). To help older adults overcome these feelings, particularly during a pandemic and quarantine, it is important to develop new communication methods such as technologies for managing and addressing the needs of the older population, technologies for managing and providing remote medical services, and technologies to encourage social engagement. Older adults who do not have access to advanced social network technologies can use more basic technologies like routine phone calls with family and friends. MAH (Mental Health America) suggests 10 structured question in order to make a brief check on the physical, social, mental and nutritional status of older adults). Moreover, volunteers and friends can visit under the Covid-19 safety regulations, or occasional visits to a park and being among other people can be helpful. Similarly, the families of older adults should receive guidance on technology, so that they can meet while social distancing. Another option is to encourage neighbors in the same building to talk to each other and find ways for mutual support.

Apart from loneliness, it is necessary to address the issue of subjective age, which has been found to rise during crises in general, and during the current quarantine, in particular. Subjective age is associated with many health outcomes. Therefore, when older adults are required to stay home as much as possible, it is imperative that policymakers take measures to relieve their loneliness and see that they have proper nutrition. One possible means for achieving these two related outcomes is to provide psychological-nutrition intervention [76] by telephone (this kind of intervention includes a weekly telephone call for about half an hour, including general talks and nutrition guidelines). This would provide human contact, as well as nutritional guidelines and encouragement to cook and eat healthy foods.

Three limitations of the current study should be noted. One is the cross-sectional study design, which does not allow for the prediction of a causal relationship between the variables. Future research should use longitudinal data to examine the relationship between feelings of loneliness, malnutrition, and subjective age. A further limitation is the use of only one question concerning loneliness. However, previous studies have also used a single question for this purpose [77]. Third, the ability to generalize the findings is limited, because the sample and the sampling procedure did not guarantee the representativeness of Jewish and Arab older adults. The sample included only older adults who answered the telephone at the moment the researchers called. Those who did not answer or did not have a telephone are not represented in this study. Fourth, all variables were measured at the same time, therefore, general attitudes and emotions may have had an influence on all ratings. These various factors may have biased the results.

Despite these limitations and the difficulty of making generalizations, the present study provides initial insights into the mechanisms of the association between loneliness, malnutrition, and subjective age during periods of imposed social isolation.

Future research could use a randomized sample that includes a personal income variable for each participant since personal income has a direct effect on nutritional intake. Another possible topic of study could address malnutrition and feelings of loneliness due to reduced hearing/vision abilities in older adults, and the difficulty of shopping and cooking on the one hand and keeping social relationships on the other hand, especially in times of crisis such as the COVID-19 pandemic when communication is very difficult due to the masks, especially for older adults with reduced vision/ hearing abilities.

## Figures and Tables

**Table 1:** Descriptive statistics of the study variables (*N* = 201).

Background Characteristics	*N*	Valid %	Mean	SD	Range
Gender					
Male	96	47.8			
Female	105	52.2			
Health status			3.61	1.15	1–5
Age			74.3	6.3	
Education			10.0	4.0	
Marital status					
No partner	54	26.9			
Has a partner	143	71.1			
Missing value	4	2.0			
Independent variable					
Loneliness feelings			2.2	1.1	1–4
Mediators					
Depressive symptoms			4.77	3.2	0–15
Malnutrition			6.82	4.3	0–20
Dependent variable					
Subjective age during COVID-19 ^1^ pandemic			2.94	1.73	0–5

^1^ Coronavirus disease.

**Table 2:** Paired *t*-tests between subjective age during the COVID-19 pandemic and subjective age before the COVID-19 pandemic (*N* = 201).

Variable	Mean	SD	*t*	*p* Value
Subjective age before COVID-19 pandemic	2.76	(1.7)	2.3	0.03
Subjective age during COVID-19 pandemic	2.94	(1.7)		

**Table 3:** Results of bivariate tests between demographic characteristics, loneliness (the independent variable), mediators and subjective age during COVID-19 pandemic (*N* = 201).

Variables		Subjective Age during the COVID-19 Pandemic			
		Mean	SD	Test ^a^	*p* Value
Demographic characteristics					
Gender	Male	2.75	1.8	*t* = −1.50	0.14
	Female	3.11	1.7		
Age				*r* = −0.05	0.52
Education				*r* = −0.08	0.29
Marital status	With partner	2.77	1.7	*t* = −2.41	0.02
	Without partner	3.43	1.68		
Independent variable					
Loneliness feelings				*r* = 0.75	0.000
Mediators					
Depressive symptoms				*r* = 0.22	0.002
Malnutrition				*r* = 0.27	0.000

^a^*t* = independent *t*-test; *r* = Pearson correlation coefficient.

**Table 4:** Summary of the mediation model analyses using 5000 bootstraps (*N* = 201).

Covariates	Independent Variable	Mediating Variable	Dependent Variable	Covariates→DV	IV→M	MV→DV	Direct Effect IV→DV	Indirect Effect		Total Effect	Adj R^2^
	(IV)	(MeV)	(DV)		(path *a*)	(path *b*)	(path *C’*)	(*a* × *b*)	95%CI	(C)	R^2^
Age group	Loneliness feelings	Depressive symptoms	Subjective age during COVID-19 pandemic	ns	0.16 **	0.27	0.27 *	0.04	−0.003–0.14	0.35 **	0.15 ***
Gender				ns							
Marital status				ns							
Education		Malnutrition		ns	0.50 *	0.08*		0.04	0.002–0.13		

Notes: Value labels of categorical variables: IV = Independent variable; M = mediator; Adj R^2^ = Adjusted R^2^; DV = Dependent variable; Age group (1 = 65–74, 2 = 75 and older); gender (1 = male, 2 = female); marital status (1 = has partner, 2 = no partner); *ns* = not significant. * *p <* 0.05. ** *p <* 0.01. *** *p <* 0.001.

## References

[1] Kotter-Grühn D., Neupert S.D., Stephan Y. (2015). Feeling old today? Daily health, stressors, and affect explain day-to-day variability in subjective age. Psychol. Health.

[2] Stephan Y., Sutin A.R., Terracciano A. (2015). How old do you feel? The role of age discrimination and biological aging in subjective age. PLoS ONE.

[3] Kornadt A.E., Hess T.M., Voss P., Rothermund K. (2018). Subjective age across the life span: A differentiated, longitudinal approach. J. Gerontol. B Psychol. Sci. Soc. Sci..

[4] Westerhof G.J., Miche M., Brothers A.F., Barrett A.E., Diehl M., Montepare J.M., Wurm S. (2014). The influence of subjective aging on health and longevity: A meta-analysis of longitudinal data. Psychol. Aging.

[5] Westerhof G.J., Barrett A.E. (2005). Age identity and subjective well-being: A comparison of the United States and Germany. J. Gerontol. B Psychol. Sci. Soc. Sci..

[6] Stephan Y., Caudroit J., Jaconelli A., Terracciano A. (2014). Subjective age and cognitive functioning: A 10-year prospective study. Am. J. Geriatr. Psychiatry.

[7] Spuling S.M., Miche M., Wurm S., Wahl H.W. (2013). Exploring the causal interplay of subjective age and health dimensions in the second half of life: A cross-lagged panel analysis. Zeitschrift Fur Gesundheitspsychologie.

[8] Ambrosi-Randić N., Nekić M., Junaković I. (2018). Felt age, desired, and expected lifetime in the context of health, well-being, and successful aging. Int. J. Aging Hum. Dev..

[9] Brothers A., Miche M., Wahl H.W., Diehl M. (2017). Examination of associations among three distinct subjective aging constructs and their relevance for predicting developmental correlates. J. Gerontol. B Psychol. Sci. Soc. Sci..

[10] Mock S.E., Eibach R.P. (2011). Aging attitudes moderate the effect of subjective age on psychological well-being: Evidence from a 10-year longitudinal study. Psychol. Aging.

[11] Keyes C.L.M., Westerhof G.J. (2012). Chronological and subjective age differences in flourishing mental health and major depressive episode. Aging Ment. Health.

[12] Shrira A., Bodner E., Palgi Y. (2014). The interactive effect of subjective age and subjective distance-to-death on psychological distress of older adults. Aging Ment. Health.

[13] Pinquart M., Sörensen S. (2001). Gender differences in self-concept and psychological wellbeing in old age: A meta-analysis. J. Gerontol. B Psychol. Sci. Soc. Sci..

[14] Choi E.Y., Kim Y.S., Lee H.Y., Shin H.R., Park S., Cho S.E. (2019). The moderating effect of subjective age on the association between depressive symptoms and cognitive functioning in Korean older adults. Aging Ment. Health.

[15] Hülür G., Hertzog C., Pearman A.M., Gerstorf D. (2015). Correlates and moderators of change in subjective memory and memory performance: Findings from the health and retirement study. Gerontology.

[16] Pearman A., Hertzog C., Gerstorf D. (2014). Little evidence for links between memory complaints and memory performance in very old age: Longitudinal analyses from the Berlin aging study. Psychol. Aging.

[17] Rippon I., Steptoe A. (2018). Is the relationship between subjective age, depressive symptoms and activities of daily living bidirectional?. Soc. Sci. Med..

[18] Segel-Karpas D., Palgi Y. (2017). “It is nothing more than a senior moment”: The moderating role of subjective age in the effect of change in memory on self-rated memory. Aging Ment. Health.

[19] Ayalon L., Palgi Y., Avidor S., Bodner E. (2016). Accelerated increase and decrease in subjective age as a function of changes in loneliness and objective social indicators over a four-year period: Results from the health and retirement study. Aging Ment. Health.

[20] Andersson L. (1998). Loneliness research and interventions: A review of the literature. Aging Ment. Health.

[21] Gierveld J.D.J. (1998). A review of loneliness: Concept and definitions, determinants and consequences. Rev. Clin. Gerontol..

[22] Hawkley L.C., Cacioppo J.T. (2010). Loneliness matters: A theoretical and empirical review of consequences and mechanisms. Ann. Behav. Med..

[23] Fees B.S., Martin P., Poon L.W. (1999). A model of loneliness in older adults. J. Gerontol. B Psychol. Sci. Soc. Sci..

[24] Shiovitz-Ezra S., Ayalon L. (2010). Situational versus chronic loneliness as risk factors for all-cause mortality. Int. Psychogeriatr..

[25] Hawkley L.C., Masi C.M., Berry J.D., Cacioppo J.T. (2006). Loneliness is a unique predictor of age-related differences in systolic blood pressure. Psychol. Aging.

[26] Perissinotto C.M., Stijacic Cenzer I., Covinsky K.E. (2012). Loneliness in older persons: A predictor of functional decline and death. Arch. Intern. Med..

[27] Cacioppo J.T., Hawkley L.C., Berntson G.G., Ernst J.M., Gibbs A.C., Stickgold R., Hobson J.A. (2002). Do lonely days invade the nights? Potential social modulation of sleep efficiency. Psychol. Sci..

[28] Wilson R.S., Krueger K.R., Arnold S.E., Schneider J.A., Kelly J.F., Barnes L.L., Tang Y., Bennet D.A. (2007). Loneliness and risk of Alzheimer disease. Arch. Gen. Psychiatry.

[29] Degges-White S., Kepic M. (2019). Friendships, subjective age, and life satisfaction of women in midlife. Adultspan J..

[30] Luo Y., Hawkley L.C., Waite L.J., Cacioppo J.T. (2012). Loneliness, health, and mortality in old age: A national longitudinal study. Soc. Sci. Med..

[31] Golden J., Conroy R.M., Bruce I., Denihan A., Greene E., Kirby M., Lawlor B.A. (2009). Loneliness, social support networks, mood and wellbeing in community dwelling elderly. Int. J. Geriatr. Psychiatry.

[32] Seeman T.E. (1996). Social ties and health: The benefits of social integration. Ann. Epidemiol..

[33] Dykstra P.A., van Tilburg T.G., Gierveld J.D.J. (2005). Changes in older adult loneliness: Results from a seven-year longitudinal study. Res. Aging.

[34] Korporaal M., Broese van Groenou M.I., van Tilburg T.G. (2008). Effects of own and spousal disability on loneliness among older adults. J. Aging Health.

[35] Wrzus C., Hänel M., Wagner J., Neyer F.J. (2013). Social network changes and life events across the life span: A meta-analysis. Psychol. Bull..

[36] Ustun T.B., Ayuso-Mateos J.L., Chatterji S., Mathers C., Murray C.J.L. (2004). Global burden of depressive disorders in the year 2000. Br. J. Psychiatry.

[37] Compton W.M., Conway K.P., Stinson F.S., Grant F.F. (2006). Changes in the prevalence of major depression and comorbid substance use disorders in the United States between 1991–1992 and 2001–2002. Am. J. Psychiatry.

[38] Fiske A., Wetherell J.L., Gatz M. (2009). Depression in older adults. Annu. Rev. Clin. Psychol..

[39] Pratt L.A., Brody D.J. (2014). Depression in the U.S. Household Population, 2009–2012 (NCHS Data Brief No. 172).

[40] Gerst-Emerson K., Jayawardhana J. (2015). Loneliness as a public health issue: The impact of loneliness on health care utilization among older adults. Am. J. Public Health.

[41] Cacioppo J.T., Hughes M.E., Waite L.J., Hawkley L.C., Thisted R.A. (2006). Loneliness as a specific risk factor for depressive symptoms: Cross-sectional and longitudinal analyses. Psychol. Aging.

[42] James B.D., Wilson R.S., Barnes L.L., Bennett D.A. (2011). Late-life social activity and cognitive decline in old age. J. Int. Neuropsychol. Soc..

[43] Lund R., Nilsson C.J., Avlund K. (2010). Can the higher risk of disability onset among older people who live alone be alleviated by strong social relations? A longitudinal study of non-disabled men and women. Age Ageing.

[44] Bekhet A.K., Zauszniewski J.A. (2012). Mental health of elders in retirement communities: Is loneliness a key factor?. Arch. Psychiatr. Nurs..

[45] Infurna F.J., Gerstorf D., Robertson S., Berg S., Zarit S.H. (2010). The nature and cross-domain correlates of subjective age in the oldest old: Evidence from the OCTO study. Psychol. Aging.

[46] Liang K. (2014). The cross-domain correlated of subjective age in Chinese oldest-old. Aging Ment. Health.

[47] Bergland A., Nicolaisen M., Thorsen K. (2014). Predictors of subjective age in people aged 40–79 years: A five-year follow-up study. The impact of mastery, mental and physical health. Aging Ment. Health.

[48] Stratton R.J., Green C.J., Elia M. (2003). Scientific criteria for defining malnutrition. Disease-Related Malnutrition: An Evidence-Based Approach to Treatment.

[49] Brownie S. (2006). Why are elderly individuals at risk of nutritional deficiency?. Int. J. Nurs. Pract..

[50] Cereda E., Pedrolli C., Klersy C., Bonardi C., Quarleri L., Cappello S., Turri A., Rondanelli M., Caccialanza R. (2016). Nutritional status in older persons according to healthcare setting: A systematic review and meta-analysis of prevalence data using MNA^®^. Clin. Nutr..

[51] De Morais C., Oliveira B., Afonso C., Lumbers M., Raats M., De Almeida M.D.V. (2013). Nutritional risk of European elderly. Eur. J. Clin. Nutr..

[52] World Health Organization (2015). World Report on Ageing and Health.

[53] Guyonnet S., Rolland Y. (2015). Screening for malnutrition in older people. Clin. Geriatr. Med..

[54] Kalan U., Arik F., Soysal P. (2019). Malnutrition in older people. Encycl. Biomed. Gerontol..

[55] Soenen S., Chapman I.M. (2013). Body weight, anorexia, and undernutrition in older people. J. Am. Med. Dir. Assoc..

[56] Chen C.C.H., Schilling L.S., Lyder C.H. (2001). A concept analysis of malnutrition in the elderly. J. Adv. Nurs..

[57] Fávaro-Moreira N.C., Krausch-Hofmann S., Matthys C., Vereecken C., Vanhauwaert E., Declercq A., Bekkering G.E., Duyck J. (2016). Risk factors for malnutrition in older adults: A systematic review of the literature based on longitudinal data. Adv. Nutr..

[58] Mangels A.R. (2018). Malnutrition in older adults: An evidence-based review of risk factors, assessment, and intervention. Am. J. Nurs..

[59] Brooks S.K., Webster R.K., Smith L.E., Woodland L., Wessely S., Greenberg N., Rubin G.J. (2020). The psychological impact of quarantine and how to reduce it: Rapid review of the evidence. Lancet.

[60] Vahia I.V., Blazer D.G., Smith G.S., Karp J.F., Steffens D.C., Forester B.P., Tampi R., Agronin M., Jeste D.V., Reynolds F.C. (2020). COVID-19, mental health and aging: A need for new knowledge to bridge science and service. Am. J. Geriatr. Psychiatry.

[61] Yesavage J.A., Brink T.L. (1983). Development and validation of a geriatric depression screening scale: A preliminary report. J. Psychiatr. Res..

[62] Hayes A.F. PROCESS [Macro]. http://afhayes.com/introduction-to-mediation-moderation-and-conditional-process-analysis.html.

[63] Preacher K.J., Hayes A.F. (2008). Asymptotic and resampling strategies for assessing and comparing indirect effects in multiple mediator models. Behav. Res. Methods.

[64] Morle J.E., Vellas B. (2020). COVID-19 and older adult. J. Nutr. Health. Aging..

[65] Graham J.E., Christian L.M., Kiecolt-Glaser J.K. (2006). Stress, age, and immune function: Toward a lifespan approach. J. Behav. Med..

[66] Foster H., Hagan J., Brooks-Gunn J. (2008). Growing up fast: Stress exposure and subjective “weathering” in emerging adulthood. J. Health Soc. Behav..

[67] Schafer M.H., Shippee T. (2010). Age identity in context: Stress and the subjective side of aging. Soc. Psychol. Q..

[68] Lazarus R.S. (1999). Stress and Emotion: A New Synthesis.

[69] Zautra A.J. (2003). Emotions, Stress, and Health.

[70] Whitbourne S.K. (2016). The Encyclopedia of Adulthood and Aging, 3 Volume Set.

[71] Eskelinen K., Hartikainen S., Nykanen I. (2016). Is loneliness associated with malnutrition in older people?. Int. J. Gerontol..

[72] Chatindiara I., Sheridan N., Kruger M., Wham C. (2020). Eating less the logical thing to do? Vulnerability to malnutrition with advancing age: A qualitative study. Appetite.

[73] Hammons A.J., Fiese B.H. (2011). Is frequency of shared family meals related to the nutritional health of children and adolescents?. Pediatrics.

[74] Locher J.L., Robinson C.O., Roth D.L., Ritchie C.S., Burgio K.L. (2005). The effect of the presence of others on caloric intake in homebound older adults. J. Gerontol. A Biol. Sci. Med. Sci..

[75] Bofill S. (2004). Aging and loneliness in Catalonia: The social dimension of food behavior. Ageing Int..

[76] Kreausukon P., Gellert P., Lippke S., Schwarzer R. (2012). Planning and self-efficacy can increase fruit and vegetable consumption: A randomized controlled trial. J. Behav. Med..

[77] Holwerda T.J., Deeg D.J., Beekman A.T., van Tilburg T.G., Stek M.L., Jonker C., Schoevers R.A. (2014). Feelings of loneliness, but not social isolation, predict dementia onset: Results from the Amsterdam Study of the Elderly (AMSTEL). J. Neurol. Neurosurg. Psychiatry.

